# Diffusion tensor MRI of the healthy brachial plexus

**DOI:** 10.1371/journal.pone.0196975

**Published:** 2018-05-09

**Authors:** Jos Oudeman, Camiel Verhamme, Maurits P. Engbersen, Mattan W. A. Caan, Mario Maas, Martijn Froeling, Aart J. Nederveen, Gustav J. Strijkers

**Affiliations:** 1 Department of Radiology, Academic Medical Center, Amsterdam, the Netherlands; 2 Department of Neurology, Academic Medical Center, Amsterdam, the Netherlands; 3 Department of Radiology, University Medical Center, Utrecht, the Netherlands; 4 Biomedical Engineering and Physics, Academic Medical Center, Amsterdam, the Netherlands; University Hospital-Eppendorf, GERMANY

## Abstract

**Introduction:**

Diffusion Tensor MRI (DT-MRI) is a promising tool for the evaluation of brachial plexus pathology. Therefore, we introduce and evaluate a fast DT-MRI protocol (8min33s scanning with 5–10 min postprocessing time) for the brachial plexus.

**Materials and methods:**

Thirty healthy volunteers within three age-groups (18–35, 36–55, and > 56) received DT-MRI of the brachial-plexus twice. Means of fractional-anisotropy (FA), mean-diffusivity (MD), axial-diffusivity (AD), and radial-diffusivity (RD) for the individual roots and trunks were evaluated. A stepwise forward approach was applied to test for correlations with age, sex, body-mass-index (BMI), bodysurface, height, and bodyweight. Within-subject, intra-rater, and inter-rater repeatability were assessed using Bland-Altman analysis, coefficient of variation (CV), intraclass-correlation (ICC), and minimal detectable difference (MDD).

**Results:**

No differences between sides and root levels were found. MD, AD, and RD correlated (*P* < 0.05) with bodyweight. Within-subject quantification proved repeatable with CVs for FA, MD, AD, and RD of 16%, 12%, 11%, and 14%, respectively.

**Discussion:**

The DT-MRI protocol was fast and repeatable. Found correlations should be considered in future studies of brachial plexus pathology.

## Introduction

Injuries and diseases affecting the peripheral nerves are conventionally assessed using nerve conduction studies [1,2]. Assessing injury or diseases at proximal locations, such as the brachial plexus is however more difficult. The roots and trunks of the plexus are located deep in the body, surrounded by other anatomical structures, hampering reliable nerve conduction studies [3]. Next to nerve conduction studies, bright mode ultrasound (US) is gaining popularity for imaging nerves. However, its use for imaging the brachial plexus is hampered by a limited penetration depth and poor contrast [4,5]. Compared to US, Magnetic resonance imaging (MRI) of the brachial plexus does not suffer from above mentioned limitations and with several new MRI neurography (MR-neurography) sequences the brachial plexus can be imaged with sufficient resolution and excellent contrast [6,7]. However, MR-neurography thus far has remained limited to imaging the nerve anatomy and subtle changes in nerve signal intensities [8]. For example, in immune mediated neuropathies the presence of hypertrophy and hyperintensity is the hallmark of the disease. However, sensitivity to detection of hypertrophy is low and hyperintensity remains difficult to assess, especially in early disease phase and atypical cases [8,9]. The latter is further complicated as the intensity along the plexus can get intensified due to the magic angle effects [10].

Diffusion-tensor MRI (DT-MRI) is a specialized MRI technique which enables quantification of the self-diffusion of water in biological tissue [11]. The technique has been particularly successful and finds widespread application in the characterization of white matter tracts in the brain. The diffusion tensor values, such as the mean diffusivity (MD) and the fractional anisotropy (FA), which reflect the average water diffusion value and its directional anisotropy have proven sensitive to pathological alterations in the brain tissue due to pathology, as can be found in stroke, tumors, and diseases that cause changes in the axonal or myelin integrity, including multiple sclerosis and other forms of neurodegeneration [12–14].

Likewise, DT-MRI can be used to characterize the peripheral nerves. The fibrous structure of the peripheral nerves can be reconstructed in 3D by fiber tractography of the principal diffusion direction [15–17]. Alterations in the structural integrity of the nerves due to disease lead to changes in the diffusion values. Such changes were already utilized to differentiate between healthy and diseased tissue, aid in diagnosis, and monitor regeneration after nerve injury [12–14,18–20]. DT-MRI therefore provides a unique, *in vivo*, and non-invasive insight in the status of the peripheral nerves.

However, DT-MRI of the brachial plexus remains challenging as the acquisition and quantitative interpretation of the diffusion characteristics of the individual roots and trunks is difficult. Before useful routine application of DT-MRI of the brachial plexus, a number of technical and methodological aspects have to be addressed. First of all, air-tissue transitions in the head and neck region may introduce magnetic field inhomogeneities, which result in image deformations and poor fat-suppression [21–23]. Moreover, it is essential to achieve sufficient signal to noise (SNR) levels for accurate and reproducible quantification [24,25], which comes at the cost of either low resolution or long scan times, hampering routine clinical implementation. The accuracy of DT-MRI measurements in the brachial plexus relies heavily on proper segmentation. As the roots and trunks are small, segmentation is often done on a single slice using anatomical scans. This approach remains difficult and time-consuming and as DT-MRI may suffer from deformations, accurate co-registration to the non-deformed anatomical scans is needed [26]. Moreover, body side as well as the level of the roots might have an influence on measurements and may complicate clinical interpretation [27,28]. Furthermore, brain diffusion values are known to correlate with sex and age [29–31] and peripheral nerve physiology and morphology differ between sexes and correlate with age, height, and bodyweight [32–34]. All together this stresses the importance to assess the dependence of brachial plexus diffusion values to these factors.

The aim of this study was therefore to introduce and evaluate a novel DT-MRI approach for the brachial plexus, which requires minimal manual user input for registration, segmentation and analysis, and thus facilitates a repeatable quantification of the diffusion characteristics. Furthermore, we investigated body side, root level differences, and studied correlations of the DT-MRI measurements with age, sex, body surface area, height, body mass index (BMI), and bodyweight.

## Methods

### Study design

We obtained written informed consent from all volunteers prior to the study. This study was waived by the local IRB as no patients were enrolled and no medical questions were answered. The subject exclusion criteria were a known injury or disease affecting the arms or neck at present or in the past and inability to undergo a MRI exam. To further exclude gross pathology affecting the roots such as disc herniation, anatomical scans were obtained and scored by a musculoskeletal radiologist with over 20 years of experience (MM). A total of 30 healthy controls were recruited of three different age groups (18–35, 36–55, and 56 and older). Each group consisted of 5 males and 5 females. Subjects were scanned twice with at least 1 hour and maximal 1 week between scan sessions.

### MRI protocol

MRI scanning was performed with a Philips Ingenia 3.0 Tesla scanner (Philips Healthcare, Best, the Netherlands). The subjects were positioned supine and the brachial plexus was covered with 16 coil elements by combining 8 elements of a 16-element body-array coil, 4 elements of the posterior part of a 16-element head array coil, and 4 elements of a spine array coil.

To reduce susceptibility artefacts caused by the air tissue transitions in the neck, a tissue susceptibility matching pillow was used in conjunction with an image-based shimming method [22]. For anatomical reference and assessing gross-pathology by the radiologist a T_2_-weighted, fat-suppressed diffusion-prepared neurography sequence was used [22,35].

The following imaging settings were used. Anatomical scan: fat-suppressed diffusion prepared three dimensional volume isotropic turbo spin echo acquisition (3D-VISTA), field of view (FOV) = 300x393x150 mm^3^, effective echo time (TE) = 61ms, repetition time (TR) = 2500 ms, turbo spin echo (TSE) factor = 100, echo spacing = 4.0 ms, voxel size = 1.1×1.1×1.1 mm^3^, receiver bandwidth = 567 Hz/pixel, fat suppression = spectral attenuated inversion recover (SPAIR), scan duration = 5min10s. DT-MRI: axial diffusion-weighted spin-echo echo-planar imaging (EPI), FOV = 288x192 mm^2^, matrix 96x62, TE = 77 ms, TR = 5969 ms, number of slices = 43, voxel size = 3x3x3 mm^3^, receiver bandwidth = 33 Hz/pixel, gradient directions = 15, b-value = 800 s/mm^2^ and 1 with b-value = 0 s/mm^2^, number of averages = 6 for all b-values, fat suppression = spectral pre-saturation with inversion recovery (SPIR) and Slice Selective Gradient Reversal (SSGR), scan duration = 8min33s. DT-MRI was scanned in an axial manner with fold-over direction in AP to optimize bandwidth and minimize distortions in the right-left direction hindering analysis. Reconstructions of the anatomical scans were made to match the orientation and x, y, and z coordinates of the DT-MRI scan. For 10 subjects the DT-MRI experiment was repeated with the radiofrequency (RF) pulses switched off to assess image noise for signal-to-noise ratio (SNR) calculations [22].

### Post-processing

DT-MRI data were processed off-line using DTItools for Mathematica 11.3 [36–39] and comprised the following steps: visual inspection of image quality, Rician noise suppression, and correction for subject motion and eddy current distortions by registering the b-value = 800 s/mm^2^ images to the b-value = 0 images with the appropriate correction of the b-matrix [40–42]. The tensor was calculated using a Weighted Least Linear Squares model. No registration to the anatomical scans was needed.

### Tractography

**Fig 1:**
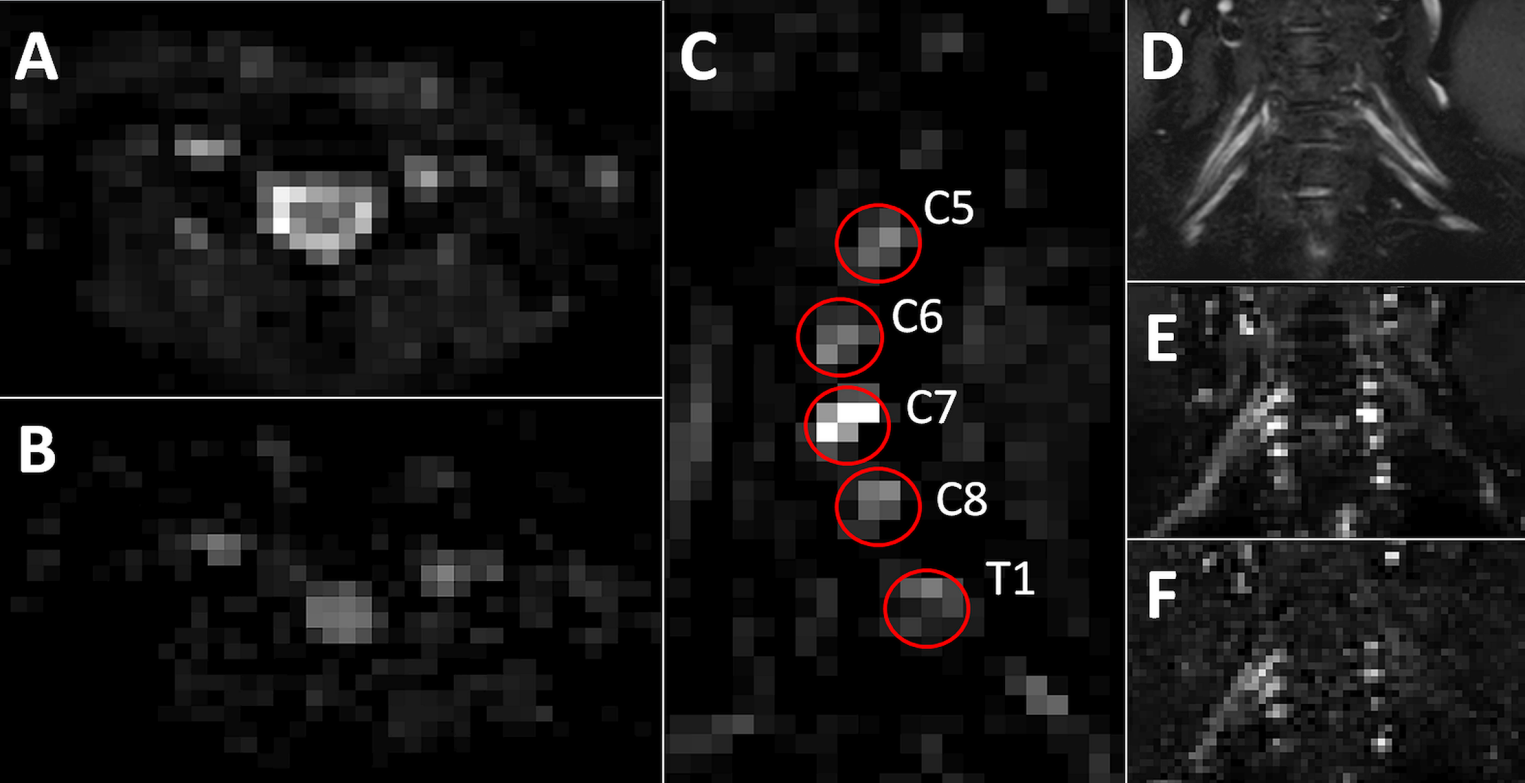
Diffusion and non-diffusion weighted images and segmentation. (A) DT-MRI with b-value = 0 in axial orientation. (B) DT-MRI with b-value = 800 s/mm^2^ in axial orientation. (C) Sagittal view of DT-MRI with b-value = 0 in which the red circles indicate the region of interest (ROI) placements per root. (D) Coronal MR neurography image with visualization of the roots and trunks. (E) DT-MRI with b-value = 0 reconstructed in coronal orientation. (F) DT-MRI with b-value = 800 s/mm^2^ reconstructed in coronal orientation.

Fiber tractography was performed using the VIST/e software [43]. Tractography was initiated from single regions of interest (ROI) in the 4 cervical roots and single thoracic root (C5-C8, Th1) per body-side, resulting in a total of 10 ROIs per subject (Fig 1). Each ROI was placed distally close to the ganglion in the sagittal plane as this area could be well distinguished on the mean diffusivity (MD) maps and the non-weighted (b-value = 0) diffusion images. A deterministic fiber tractography algorithm with a step size of 0.15 voxel initiated from the ROI with a seed density of 1 per mm^2^. Stopping criteria for the tractography included a minimal Fractional Anisotropy (FA) of 0.1 and maximum of 0.8, as well as a maximum angle change of 14 degrees per step. Furthermore, only fibers with a minimum length of 3 cm were reconstructed. To assess inter- and intra-observer agreement all ROIs were placed twice by the same observer and once by a second observer (JO and ME).

### Tract analysis

**Fig 2:**
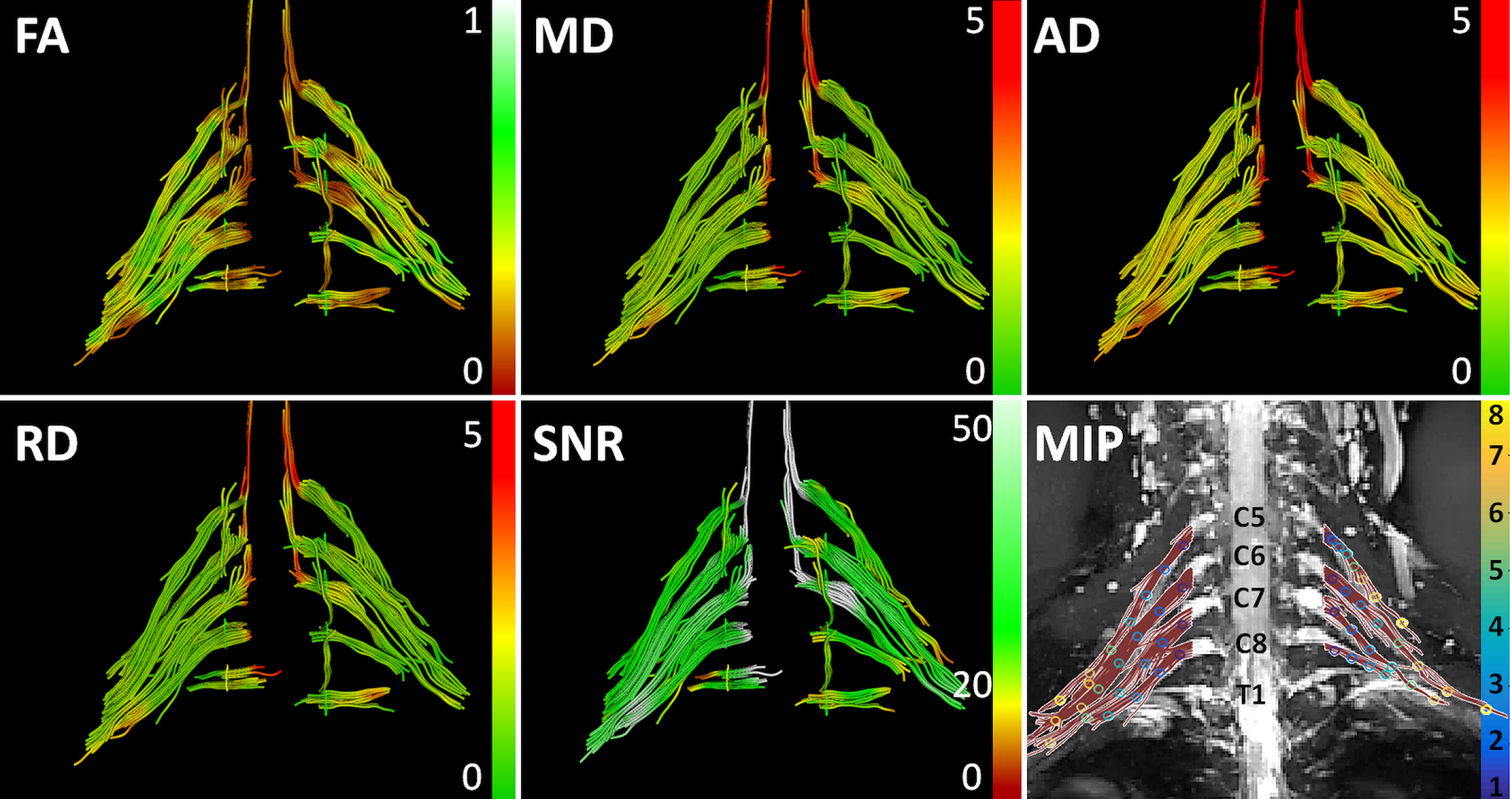
DT-MRI tractography. Representative example of a DT-MRI tractography dataset (64 years old male), including tractography color coded for fractional anisotropy (FA), mean diffusivity (MD), axial diffusivity (AD), radial diffusivity (RD) and signal to noise (SNR). The bottom right image is a maximum intensity projection (MIP) of the brachial plexus neurography image (MR-Neurography) with superimposed fiber tracts of the postganglionic tissue only. The circles along the tracts indicate the sample sections which are color-coded for sample number.

Next, the diffusion values along the reconstructed fibers were quantified using a custom-built toolbox for Matlab [4]. This step further decreases measurement errors by selecting only fibers belonging to the roots and trunks, this is done by taking samples along the complete length of the average path of all reconstructed fibers thereby avoiding the inclusion of mainly shorter fibers close to the seed and this also excludes any aberrant fibers not following the direction of the roots and trunks. Fibers were cut just distally from the ganglia and proximally to Erb’s point (Fig 2). Because of inconsistent fiber tractography, the fibers in Th1 were not considered further.

The roots and trunks were automatically divided in equally sized nerve sections and sampled accordingly (8 samples for C5 and C6, 6 for C7, and 4 for C8). In each sample, the mean of the fractional anisotropy (FA), mean diffusivity (MD), axial diffusivity (AD) and radial diffusivity (RD) were calculated. From the 10 datasets intended for this purpose, SNR of the non-weighted (b-value = 0) acquisition was calculated over the roots and trunks using the same methods.

### Statistics

Patient characteristics are presented as the median with range per age category. DT-MRI values (FA, MD, AD, and RD) of the first and second scan were averaged and presented as mean with standard deviation (SD). Right-left differences and differences between the roots of C5 to C8 were assessed using a repeated-measures mixed-model ANOVA with the side and level of the root within one subject being the repeated measure. Within this model we used a stepwise forward approach, to identify one by one the possible covariates being age (years), sex, BMI (kg/m^2^), body surface (m^2^), height (m), and bodyweight (kg) (P<0.05). This approach enables to test dependent covariates (being BMI, body surface, height, and bodyweight) separately and identifies the highest likely contributor of a covariate or a dependent part of it, which can be added to the model. Mauchly’s test for sphericity was performed and in case the sphericity assumption was violated a Greenhouse-Geisser correction was applied.

For assessing repeatability, the within-subject (comparisons of the first scan session for the first observer who analyzed the data twice), intra-rater (comparisons of the first scan session for the first observer who analyzed the data twice), inter-rater (comparisons of the first scan session between the first and second observer) variability of the mean per root and right-left differences was determined using Bland-Altman analysis. In the Bland-Altman plots the mean difference and the 95% confidence interval (1.96*SD) are shown. Within-subject coefficients of variation (CV) were calculated for each nerve separately. The CV is calculated as CV = 100% * SD _paired difference_ / Mean.

The minimal detectable difference (MDD) was estimated as a measure for the responsiveness of DT-MRI [44]. Responsiveness is defined here as the ability of the outcome measure to detect clinically relevant changes within an individual patient. The MDD is calculated as MDD = 1.96*SD_paired difference_ [44].

Inter- and intra-observer agreement were assessed using the Intra Class Coefficients (ICC) for absolute agreement and single measures, with ICC<0.40 considered poor, 0.40–0.59 fair, 0.60–0.74 good, and >0.74 considered excellent agreement. In case of a significance of one or more covariates found using the repeated measures ANOVA this was further explored using linear regression.

## Results

All but one subject were scanned twice. One subject (male, group 36–55 years old) did not undergo the second scan and was therefore left out of the reproducibility analysis. Subject characteristics can be found in Table 1 for the subjects in the 3 age groups. There was a large range for height, bodyweight, and BMI, but there were no statistical differences between groups for any of these characteristics. Furthermore, radiological assessment revealed no pathology potentially affecting the brachial plexus.

**Table 1:** Group characteristics.

	Group 1 (5M, 5F)		Group 2 (5M, 5F)		Group 3 (5M, 5F)	
Age (years)	27	(24–33)	42	(35–55)	63	(57–83)
Height (m)	1.76	(1.68–1.96)	1.76	(1.59–1.83)	1.72	(1.56–1.92)
Weight (kg)	71	(60–80)	74	(47–95)	75	(53–103)
BMI (kg/m^2^)	22.4	(20.5–25.9)	25.5	(16.7–32.1)	24.0	(20.2–32.0)

Group characteristics reported as the median and the range (minimum—maximum). M = male, F = female.

All MR images were successfully acquired and visually checked for artifacts potentially hindering analysis. No data needed to be discarded because of artifacts. Fig 1 shows a representative dataset with non-weighted (b-value = 0) (Fig 1A), and diffusion-weighted (b-value = 800 s/mm^2^) (Fig 1B) axial images. Fig 1C shows a sagittal reconstruction of the non-weighted (b-value = 0) images with the positions of the tractography seeding ROIs for the roots. In Fig 1D–1F a coronal MR-Neurography image (Fig 1D) is shown above non-weighted (b-value = 0) (Fig 1E) and diffusion-weighted (b-value = 800 s/mm^2^) (Fig 1F) coronal reconstructions, in which the anatomical correspondence can be appreciated.

Tractography was successful for all C5-C8 nerves. However, in 30 datasets (52%) the fibers for Th1 were not reconstructed all the way to Erb’s point. Therefore, measurements of Th1 were not considered for further analysis. Fig 2 shows representative results of the fiber tractography in which the fibers were color coded for FA, MD, AD, RD, and SNR. The bottom right panel in Fig 2 is a maximum intensity projection (MIP) of the MR neurography dataset with fibers superimposed, demonstrating excellent co-registration of both types of nerve depictions. This also shows the total lengths of the trunk of the nerves which were considered and it contains the sample points (8 sample points for C5 and C6, 6 for C7, and 4 for C8) used for quantitative analysis. The tractography-based segmentation took about 5–10 minutes per dataset. The total time for the post-processing, including importing and exporting of the data in the DTItools, VIST/e, and Matlab toolboxes amounted up to 1 hour per dataset. From this whole process, user interaction was needed for segmentation and defining were to cut the fibers to exclude the ganglia and myelum.

The average SNR of the non-weighted images for all C5 to C8 nerves was 21 ± 8. Mean values for FA, MD, AD and RD are given in Table 2. Significant correlations for MD (*P* = 0.005), AD (*P* = 0.002), and RD (*P* = 0.013) with bodyweight were found. Although the diffusion parameters also correlated significant with BMI, these were less strong (*P* < 0.04). No correlations with height and body surface area were found. Therefore, only bodyweight was added to the model as a covariate. After correction for bodyweight no statistical right-left differences or differences between the levels of the roots were found.

**Table 2:** DT-MRI values and repeatability indices.

	FA				MD (x 10^−3^ mm^2^/s)				AD (x 10^−3^ mm^2^/s)				RD (x 10^−3^ mm^2^/s)			
	Mean ± SD	CV (%)	MDD	RL diff (%)	Mean ± SD	CV (%)	MDD	RL diff (%)	Mean ± SD	CV (%)	MDD	RL diff (%)	Mean ± SD	CV (%)	MDD	RL diff (%)
**Right**																
**C5**	0.30±0.02	15.7	0.09		1.38±0.13	15.7	0.29		1.81±0.16	10.2	0.37		1.16±0.12	12.2	0.28	
**C6**	0.33±0.03	10.7	0.07		1.38±0.09	10.7	0.22		1.89±0.11	8.0	0.30		1.13±0.09	9.3	0.21	
**C7**	0.34±0.03	14.3	0.10		1.34±0.10	14.3	0.32		1.84±0.12	11.4	0.41		1.09±0.09	14.1	0.30	
**C8**	0.33±0.05	23.8	0.15		1.29±0.18	23.8	0.44		1.74±0.21	17.1	0.58		1.07±0.17	19.3	0.40	
**Total**	0.32±0.05	16.7	0.11		1.37±0.16	12.4	0.32		1.83±0.18	11.9	0.42		1.14±0.16	14.0	0.30	
**Left**																
**C5**	0.32±0.03	13.5	0.08		1.36±0.14	12.0	0.31		1.82±0.17	11.5	0.41		1.12±0.14	13.2	0.29	
**C6**	0.34±0.03	14.0	0.09		1.36±0.09	8.0	0.21		1.88±0.10	9.0	0.33		1.10±0.09	9.0	0.20	
**C7**	0.35±0.03	15.6	0.11		1.36±0.08	12.2	0.33		1.88±0.10	11.2	0.41		1.10±0.08	14.6	0.32	
**C8**	0.33±0.04	14.9	0.10		1.41±0.18	13.2	0.37		1.90±0.21	11.2	0.42		1.17±0.17	15.7	0.36	
**Total**	0.33±0.03	14.5	0.10		1.37±0.13	11.8	0.32		1.87±0.16	11.0	0.40		1.12±0.12	13.7	0.30	
**combined**																
**C5**	0.31±0.03	14.5	0.09	-5.9±9.2	1.37±0.14	11.5	0.30	1.6±10.2	1.81±0.16	11.1	0.39	-0.3±9.5	1.14±0.12	12.8	0.29	3.2±11.1
**C6**	0.33±0.03	12.4	0.08	-1.3±6.6	1.37±0.09	8.0	0.21	-1.2±5.8	1.88±0.11	8.5	0.31	0.8±5.9	1.11±0.09	9.1	0.20	1.6±6.4
**C7**	0.34±0.03	15.1	0.08	-1.9±7.4	1.35±0.09	12.2	0.22	-1.6±7.2	1.86±0.11	11.2	0.31	-2.3±6.2	1.09±0.09	14.4	0.20	-1.0±8.5
**C8**	0.33±0.05	19.9	0.12	-0.7±13.6	1.35±0.19	15.5	0.41	-8.7±12.1	1.82±0.22	14.3	0.51	-8.6±11.7	1.12±0.18	17.7	0.38	-8.8±13.3
**Total**	0.33±0.04	15.6	0.10	-2.1±9.8	1.36±0.13	12.1	0.32	-1.9±9.9	1.85±0.16	11.4	0.41	-2.6±9.3	1.12±0.13	13.9	0.30	-1.2±11.1

Mean values and standard deviation (SD) of fractional anisotropy (FA), mean diffusivity (MD), axial diffusivity (AD), and radial diffusivity (RD) per nerve, per side (right-left), and combined. Furthermore, the coefficient of variation (CV) and minimal detectable difference (MDD) are shown. Right-left differences are presented as a percentile difference (RL diff).

**Fig 3:**
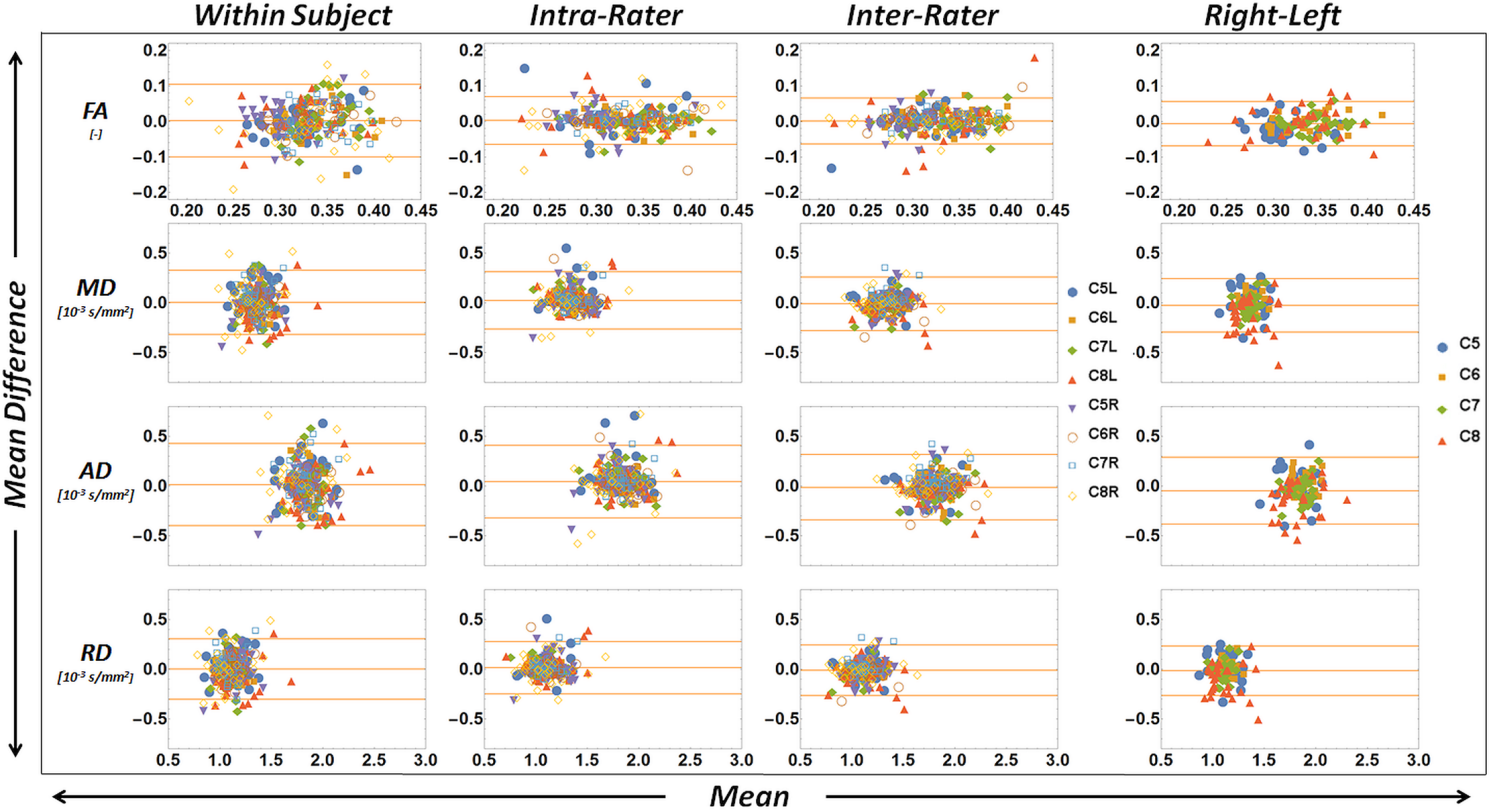
Bland-Altman analysis of DT-MRI derived diffusion values. Bland-Altman analysis of DT-MRI derived diffusion values FA, MD, AD, and RD. (Column 1) Within-subject variability. Comparisons between the first and second scan session for the first observer. (Column 2) Intra-rater variability. Comparisons of the first scan session for the first observer who analyzed the data twice. (Column 3) Inter-rater variability. Comparisons of the first scan session between the first and second observer. (Column 4) Data of the first scan session analyzed for right-left differences. The top and bottom orange lines are the 95% confidence interval (CI) of the measurements. The middle orange line indicates the mean difference between the measurements.

Bland-Altman analysis for within-subject, intra-observer and inter-observer variability are shown in Fig 3 for FA, MD, AD, and RD. Furthermore, CVs ranged between 10.7% to 23.8% for FA, 8.0% to 15.5% for MD, 8.0% to 17.1% for AD, and 9.1% to 19.3% for RD. Values per nerve can be found in Table 2. The root and trunk of C6 had the best and the one of C8 had the worst within-subject reproducibility. The MDD was 0.10 for FA, 0.32 x 10^−3^ mm^2^/s for MD, 0.41 x 10^−3^ mm^2^/s for AD and 0.30 x 10^−3^ mm^2^/s for RD. ICC values for intra-observer were all excellent with 0.753 for FA, 0.790 for MD, 0.740 for AD and 0.816 for RD. Inter-observer ICCs were all good being 0.732 for FA, 0.650 for MD, 0.644 for AD and 0.666 for RD. All data can be found in S1 Dataset and S2 Dataset.

**Fig 4:**
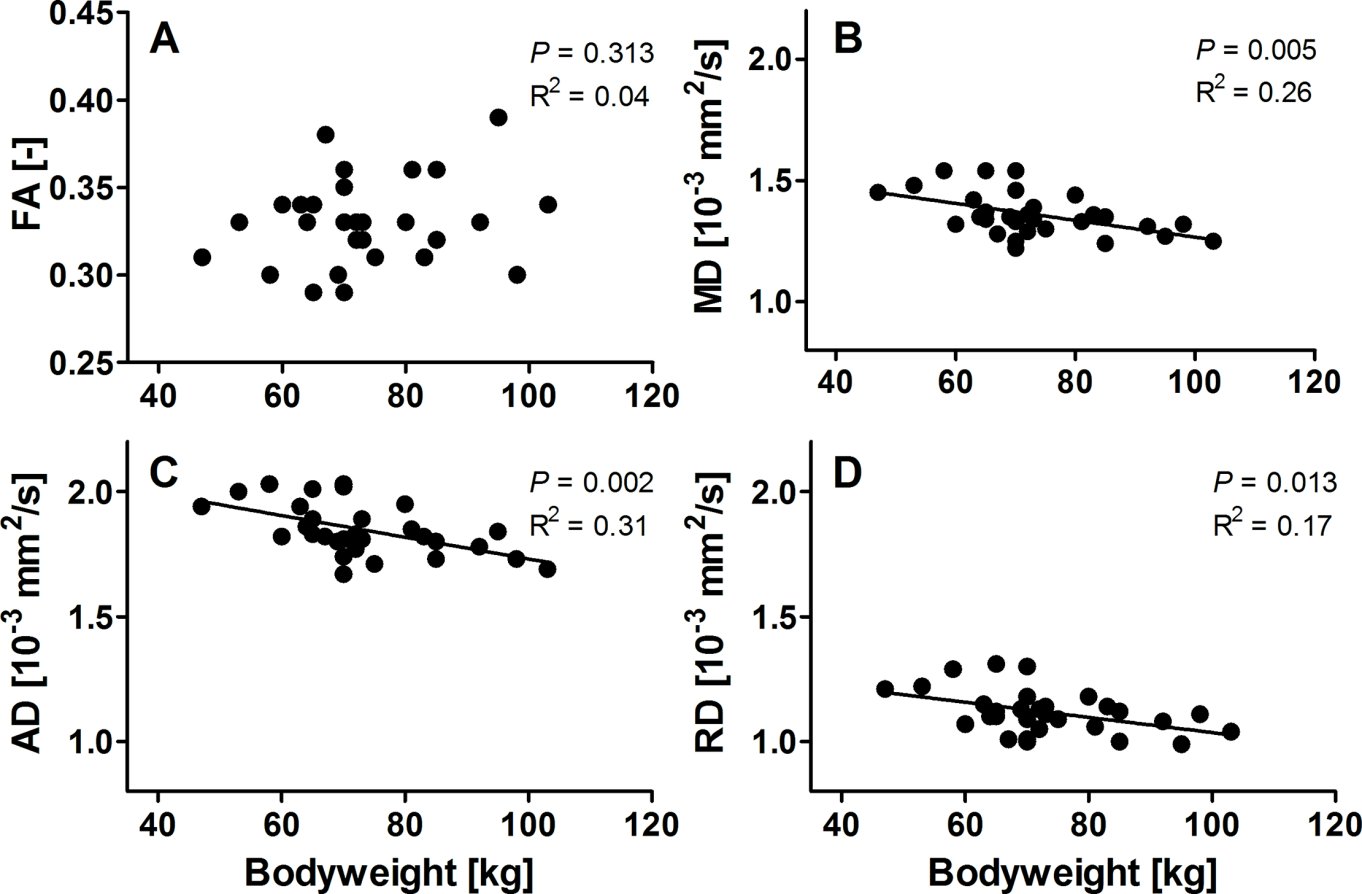
Scatterplots of diffusivity values as a function of bodyweight. Scatterplots of fractional anisotropy (FA), mean diffusivity (MD), axial diffusivity (AD), and radial diffusivity (RD) as function of bodyweight. Regression lines are linear fits for MD (P = 0.005), AD (P = 0.002), and RD (P = 0.013).

Since the nerve FA, MD, AD, and RD values of C5-C8 were not statistically different, we averaged values over all roots per subject to perform linear regression with bodyweight. As mentioned above we did not find associations of diffusion values with age, sex, body surface, or height and therefore did not perform additional linear regression for these characteristics. Although BMI seemed correlated, it was the bodyweight which defined this correlation and therefore also BMI was not considered. The correlation with bodyweight was significant for MD (*P* = 0.005), AD (*P* = 0.002), and RD (*P* = 0.013), but not for FA, as shown in Fig 4. Linear regression resulted in a slope in MD of -3.49 x 10^−6^ mm^2^/s per kg bodyweight with an intercept of 1.62 x 10^−3^ mm^2^/s and R^2^ = 0.26. For AD, the slope was -4.40 x 10^−6^ mm^2^/s per kg bodyweight with an intercept of 2.17 x 10^−3^ mm^2^/s and R^2^ = 0.31. Finally, for RD regression resulted in a slope of -3.03 x 10^−6^ mm^2^/s per kg bodyweight with an intercept of 1.34 x 10^−3^ mm^2^/s and R^2^ = 0.17.

## Discussion

In this paper, we have presented a DT-MRI scanning protocol and post-processing approach for the brachial plexus. The post-processing of the data, which included diffusion registration and segmentation of the roots C5-C8 and Th1, was semi-automatic and could be done in less than 10 minutes of user interaction. We tested repeatability of the acquisition and processing methods in a cohort of healthy subjects. This resulted in an adequate within-subject, intra-rater, and inter-rater variation. The cohort had a large spread in age, BMI, height, and weight, which enabled us to assess correlations of the DT-MRI parameters with these subject characteristics.

The scan duration of the DT-MRI sequence was below 10 min, which was well tolerated by the volunteers. This scan time is comparable to the one required for standard 3D-STIR (short tau inversion recovery) anatomical scanning of the plexus. Also, our DT-MRI protocol has no further requirements with respect to patient positioning and is performed during free-breathing. We therefore anticipate that this protocol will be acceptable for patients with brachial plexus pathology, which is supported by initial experiences in an ongoing study.

A relatively large and isotropic voxel-size was chosen to avoid anisotropy bias [45] and guarantee sufficient SNR (typically >15 is required), which is needed for accurate diffusion parameter estimations and tractography [24,25]. This voxel-size facilitated an adequate bandwidth, limiting geometrical distortions in the EPI readout. Additionally, we used a low-cost susceptibility matching pillow for improved field homogeneity [22]. Taken together, this enabled accurate DT-MRI reconstructions of the nerves that matched the MNR images all the way up to Erb’s point except for Th1. For Th1 we believe the location close to the apex of the lungs introduced movements as well as susceptibility artifacts resulting in poor SNR, poor fat-suppression, and consequently poor fiber tractography.

Our approach allows for segmentation of the nerves using the DT-MRI tractography instead of a challenging segmentation based on image contrast between the nerves and its surrounding tissue. Furthermore, no challenging registration between the DT-MRI data and anatomical scans is needed. We used the MD maps and non-weighted (b-value = 0) images for drawing the seed ROIs as the roots could be easily depicted in the sagittal planes. Subsequently, automatic fiber tractography was sufficient for segmentation of the nerves. This ensured that only nerve tissue was included in the quantitative diffusion parameter analysis and that partial volume with neighboring muscle and fat tissues was avoided as much as possible. Subsequently, we used a clustering method for tracts as described by Caan *et al*. [46] in which measured clusters are spread evenly along the arc length and the mean is evenly weighted over the root and trunk as a whole. By doing so, outlier fibers were excluded from the averaged diffusion parameters over the tract.

A potential risk for the reliability and sensitivity are partial volume effects in which the neighboring fat and muscle tissue are included in the voxels identified as nerve tissue [45]. We have tried to mitigate these effects as much as possible using the following strategies. First, the use of b-values of 800 mm^2^/s reduces the signal coming from muscle tissue considerably. Secondly, we have simultaneously applied two fat-suppression techniques (SPIR and SSGR) to get rid of fat contamination in the acquisition [47]. Thirdly, as described above, we quantified the diffusion values along the average fiber path in the nerves, which runs in the middle of the nerve and is mostly unaffected by partial volume effects. Additionally, we found no correlation with root diameter and the diffusion parameters, providing strong evidence for the absence of strong partial volume effects.

Our approach to segmentation and analysis is fast compared to other protocols in the literature which try to assess the full extent of the roots and trunks. For example, in a study by Tagliaflico *et al*. on average 47 manually drawn ROIs per root were needed for analysis [27], which takes much longer than drawing a single ROI per root for our method. Still, we found that the repeatability of our protocol was almost identical compared to the study by Tagliafico *et al*. [27] with CVs ranging from 11–24% for FA and 8–17% for MD, compared to 6–20% for FA and 6–18% for the MD in our study. Importantly, our method proved equally rater dependent for MD and less for FA.

The DT-MRI values found in our study compare well with other studies of the brachial and sacral plexus [17,19,27,48]. However, FA was slightly lower compared to Tagliafico *et al*. [27]. This difference in FA might be caused by differences in SNR values as FA is most prone to overestimation by low SNR. MD on the contrary is relatively independent of SNR [24,25]. In another study by Ho *et al*. values for both FA and MD are slightly higher, but these authors sample solely the most proximal part of the roots which make comparing the results difficult [49].

For clinical application, identification of injured or diseased roots or trunks comparison with the contralateral healthy root or trunk has been suggested [27,50]. Our results suggest this approach can be useful as we found no significant difference between right and left. However, it should also be used with care as we observed relatively high right-left differences in individual subjects which amounted up to 19% for FA, 19% for MD, 20% for AD, and 21% for RD (95% CI). Interestingly, these variations are similar to the intra-rater variability suggesting that the observed right-left differences are within the measurement repeatability rather than originating from actual physiological differences, such as left or right handedness.

Whether the observed variability is sufficiently low to detect subtle pathological changes in individuals remains to be seen. For example, in a study by Chen *et al*. compressed nerves were compared to unaffected contralateral ones [50]. Mean differences of 15% for FA, 13% for MD, 8% for AD, and 16% for RD were found, which lie within measurement variability and therefore would lead to a significant number of false negatives. Two studies reported differences between healthy nerves and those affected by CIDP of 19–30% for FA and 0–12% for MD [14,18], indicating that diagnosis of individual patients may still be hampered by the CI of within-subject variation. However, studies on diffusion parameters of peripheral nerves are still scarce and more research of various pathological cases in the context of additional clinical data is needed to establish benefit for the individual patient. In any case, DT-MRI might be a powerful tool to study pathophysiology of immune mediated diseases, for follow up in case of injury, in treatment studies, and studies of the natural course of disease [12,18,20,51,52].

In the current literature, tractography is presented as a valuable tool for assessing morphological abnormalities, i.e. nerve continuity, by some investigators [19,48,53]. However, we believe that DT-MRI and tractography provide suitable methods to investigate diffusional changes in the tissue caused by pathology, but other high-resolution MRI sequences are more suitable to assess morphological abnormalities. For example, the MR neurography sequence used in this study is a 3D T_2_-weighted fat-suppressed sequences with a much higher spatial (isotropic) resolution than the SE-EPI used for DT-MRI [35,54]. Furthermore, these MR-neurography sequences provide excellent contrast of the nerves with the surrounding tissues and do not suffer from geometrical distortions. It also allows reconstruction of MIPs [7,22] and can provide clinicians with reformatted images in all desired orientations [55,56]. With these techniques the somatotopy of brachial plexus could even be revealed, which is currently impossible with the current spatial resolutions of DT-MRI sequences and derived tractographies [57].

Interestingly, we found a significant correlation of bodyweight with MD, AD, and RD, but not with FA. A possible cause could be residual and inadequately suppressed fat [58,59]. Residual fat would indeed lead to a decrease of MD, AD, and RD but one would expect a stronger correlation with BMI in this case and also an increase in FA with bodyweight or BMI is expected [58,59]. Significant correlations of cross-sectional areas (CSA) and conduction velocities with bodyweight and height were reported based on US and NCS measurements [34,60], but no explanations were provided. Our findings are supported by a recent DT-MRI study of the peripheral nerves in which also a strong correlation between bodyweight and diffusion parameters was found. Interestingly this study also found a strong inverse correlation between age and FA, which was absent in our data. The authors suggest the reason for this age dependency is the result of an increased RD and decreased AD as a result of axonal neuropathy and myelin sheath damage. This might also explain the absence of this correlation in our study as neuropathic changes are mostly seen in the periphery [61].

We are not aware of other MRI studies correlating bodyweight to the characteristics of brachial plexus roots and trunks. The origin of this correlation therefore remains unknown and more research is warranted. Importantly, this correlation needs to be considered as a confounder in clinical studies in the brachial plexus using DT-MRI parameters as readout.

## Conclusions

In this study, we evaluated a DT-MRI protocol for quantification of brachial plexus diffusion values. The DT-MRI protocol enabled repeatable quantification of brachial plexus diffusion values in a clinical feasible scan time and only limited manual input. We found a strong and significant correlation of bodyweight with MD, AD, and RD. This correlation needs to be taken into account as a confounder in clinical studies in the brachial plexus using DT-MRI parameters as readout.

## Supporting information

S1 Dataset Correlations. (XLSX)

S2 Dataset Reproducibility. (XLSX)
